# Transplantation of cultured dental pulp stem cells into the skeletal muscles ameliorated diabetic polyneuropathy: therapeutic plausibility of freshly isolated and cryopreserved dental pulp stem cells

**DOI:** 10.1186/s13287-015-0156-4

**Published:** 2015-09-07

**Authors:** Masaki Hata, Maiko Omi, Yasuko Kobayashi, Nobuhisa Nakamura, Takahiro Tosaki, Megumi Miyabe, Norinaga Kojima, Katsutoshi Kubo, Shogo Ozawa, Hatsuhiko Maeda, Yoshinobu Tanaka, Tatsuaki Matsubara, Keiko Naruse

**Affiliations:** Department of Removable Prosthodontics, School of Dentistry, Aichi Gakuin University, Nagoya, Japan; Department of Internal Medicine, School of Dentistry, Aichi Gakuin University, Nagoya, Japan; Department of Oral Pathology, School of Dentistry, Aichi Gakuin University, Nagoya, Japan

## Abstract

**Introduction:**

Dental pulp stem cells (DPSCs) are mesenchymal stem cells located in dental pulp and are thought to be a potential source for cell therapy since DPSCs can be easily obtained from teeth extracted for orthodontic reasons. Obtained DPSCs can be cryopreserved until necessary and thawed and expanded when needed. The aim of this study is to evaluate the therapeutic potential of DPSC transplantation for diabetic polyneuropathy.

**Methods:**

DPSCs isolated from the dental pulp of extracted incisors of Sprague–Dawley rats were partly frozen in a −80 °C freezer for 6 months. Cultured DPSCs were transplanted into the unilateral hindlimb skeletal muscles 8 weeks after streptozotocine injection and the effects of DPSC transplantation were evaluated 4 weeks after the transplantation.

**Results:**

Transplantation of DPSCs significantly improved the impaired sciatic nerve blood flow, sciatic motor/sensory nerve conduction velocity, capillary number to muscle fiber ratio and intra-epidermal nerve fiber density in the transplanted side of diabetic rats. Cryopreservation of DPSCs did not impair their proliferative or differential ability. The transplantation of cryopreserved DPSCs ameliorated sciatic nerve blood flow and sciatic nerve conduction velocity as well as freshly isolated DPSCs.

**Conclusions:**

We demonstrated the effectiveness of DPSC transplantation for diabetic polyneuropathy even when using cryopreserved DPSCs, suggesting that the transplantation of DPSCs could be a promising tool for the treatment of diabetic neuropathy.

**Electronic supplementary material:**

The online version of this article (doi:10.1186/s13287-015-0156-4) contains supplementary material, which is available to authorized users.

## Introduction

Dental pulp stem cells (DPSCs), which are mesenchymal stem cells located in the dental pulp cavity [1, 2], are considered a possible cell source for regenerative medicine since DPSCs can be obtained from extracted teeth without an additional invasion, such as from wisdom teeth or premolars extracted for orthodontic reasons. DPSCs are highly proliferative and can differentiate into osteoblasts, odontoblasts, adipocytes, and neuronal cells [3, 4]. The clinical application of DPSCs is ongoing, such as for bone regeneration [5–7] and spinal cord injury [8].

Diabetic polyneuropathy (DPN) is the most common and early developing complication in both type 1 and type 2 diabetes [9]. Its various symptoms not only reduce the quality of life, but the existence of DPN affects mortality [10]. Although recent anti-symptomatic drugs are effective for diabetic nerve pain [11], a more effective treatment is needed to cure DPN.

We suggest that progenitor/stem cell transplantation is a potentially useful therapy for the treatment of DPN since it improves the nerve blood flow and nerve conduction velocity as well as the sensory disorder in diabetic animals [12–14]. However, previous investigations determined that the impairment of cell function by diabetes occurs in progenitor/stem cells [15–17]. Aging also impairs progenitor/stem cells [18, 19]. Recently, we demonstrated that the therapeutic efficacy of bone marrow-derived mononuclear cells (MNCs) for DPN was impaired in MNCs from aged rats compared with those from young rats [20].

DPSCs could be isolated from the teeth extracted by for orthodontic reasons in people by their 20s. If DPSCs isolated from young teeth can be cryopreserved for future use, some of the problems of diabetes and aging may be treated. In this paper, we investigated whether transplantation of DPSCs can improve DPN. Furthermore, to investigate the viability of cryopreserved DPSCs (cryo-DPSCs), we compared the proliferative and differential ability of freshly isolated DPSCs (fresh-DPSCs) and cryo-DPSCs and examined their therapeutic potential in DPN. We demonstrated that the transplantation of fresh-DPSCs into hindlimb skeletal muscles ameliorated the sciatic nerve blood flow and sciatic nerve conduction velocity in association with the increased capillary to muscle ratio and intra-epidermal nerve fiber density in diabetic rats. The cryo-DPSCs showed equivalent ability for proliferation and differentiation compared with the fresh-DPSCs. Cryo-DPSC transplantation showed equivalent efficacy for improving the nerve conduction velocity and nerve blood flow in diabetic rats.

## Methods

### Isolation of rat DPSCs

Dental pulp was harvested from the extracted incisors of 6-week-old male Sprague–Dawley rats (Chubu Kagakushizai, Nagoya, Japan) or green fluorescent protein (GFP)-transgenic SD rats (SD-Tg(CAG-EGFP)Cz-0040sb; Japan SLC, Inc., Hamamatsu, Japan). DPSCs were isolated from the dental pulp by digestion with phosphate-buffered saline containing 0.1 % collagenase and 0.25 % trypsin. DPSCs were cultured with alpha modification of the Eagle’s medium (α-MEM; GIBCO, Billings, MT, USA) supplemented with 20 % fetal bovine serum embryonic stem cell-qualified (GIBCO) [21]. DPSCs from passage 3 to 6 were used for all experiments. All experimental protocols were conducted according to the Regulations for Animal Experiments in Aichi Gakuin University, and were approved by the Institutional Animal Care and Use Committees of Aichi Gakuin University.

### Detection of cell surface markers

DPSCs at passage 3 were subjected to flow cytometric analysis (FACS Calibur; Becton Dickinson, Franklin Lakes, NJ, USA). Cells were stained with the FITC-conjugated mouse monoclonal antibody against rat CD90, the FITC-conjugated hamster antibody against rat CD29, and PE-conjugated mouse monoclonal antibodies against rat CD34, CD49d, and CD45 (Becton Dickinson). Isotype-identical antibodies served as controls. Data were analyzed with CELLQUEST software (Becton Dickinson) [22].

### Animals and induction of diabetes

Six-week-old male Sprague–Dawley rats were provided by Chubu Kagakushizai. Diabetes was induced by the intraperitoneal injection of streptozotocine (STZ; 60 mg/kg; Sigma Chemical, St. Louis, MO, USA). Control rats received an equal volume of citric acid buffer.

### Transplantation of DPSCs

Eight weeks after the induction of diabetes, 1 × 10^6^ cells/limb of DPSCs in 1.0 ml saline were injected into 10 points in the unilateral hindlimb skeletal muscles of the normal and diabetic rats. Vehicle (saline) was injected into the hindlimb muscles of the other side as the control. Four weeks after the transplantation, the following measurements were performed.

### Sciatic nerve conduction velocities

Rats were anesthetized with isopentane and placed on a heated pad in a room maintained at 25 °C to ensure a constant rectal temperature of 37 °C. Motor nerve conduction velocity (MNCV) between the ankle and sciatic notch, and sensory nerve conduction velocity (SNCV) in the sciatic nerve between the ankle and knee were measured using a Neuropak NEM-9400 instrument (Nihon-Koden, Osaka, Japan).

### Sciatic nerve blood flow

Sciatic nerve blood flow (SNBF) was measured by laser-Doppler flowmeter (FLO-N1, Omegawave, Tokyo, Japan). The skin of an anesthetized rat was cut along the femur and then an incision through the fascia was carefully made to expose the sciatic nerve. The blood flow was measured by a laser-Doppler probe placed 1 mm above the nerve.

### Tissue collection

Rats were killed by an overdose of pentobarbital. The soleus muscles and footpads from both sides were obtained from the normal and the diabetic rats. Extracted tissues were fixed by 4 % paraformaldehyde overnight, and then embedded in paraffin.

### Capillary number to muscle fiber ratio

The soleus muscles in paraffin were cut into 5-μm sections for immunohistochemical staining. The sections were incubated overnight at 4 °C with the primary antibody (anti-vWF polyclonal antibody; DAKO Japan, Tokyo, Japan) diluted 1:600 and subsequently stained using the Simplestain rat system (Nichirei, Tokyo, Japan) according to the manufacturer’s instructions. The negative control was performed by omitting the anti-vWF antibody.

The sections were also stained with the primary antibody (anti-PECAM-1 polyclonal antibody; Santa Cruz Biotechnology Inc., Dallas, TX, USA) diluted 1:200 and visualized using Alexa fluor 594 anti-rabbit secondary antibody (Invitrogen, Carlsbad, CA, USA). Nuclei were stained with 4’-6-diamidino-2-phenylindole (DAPI). The capillary to muscle ratio was counted under a fluorescence microscope (×200).

### Location and differentiation of transplanted DPSCs in skeletal muscles

To investigate the survival and the differentiation of transplanted DPSCs, DPSCs isolated from GFP rats were transplanted into the hindlimb skeletal muscles of diabetic rats. Four weeks after the transplantation, the DPSC-transplanted skeletal muscles were excised and fixed in 4 % paraformaldehyde. The specimens were embedded within an OCT compound (Sakura Finetechnical, Tokyo, Japan) and cut into 5-μm sections. The sections were stained with the primary antibody against PECAM-1 (1:400), FABP (1:100), osteocalcin (1:100) (R&D Systems, Minneapolis, MN, USA), neuronal nuclei (NeuN) (1:400; Millipore, Billerica, MA, USA), and glial fibrillary acidic protein (1:400; Santa Cruz). After washing, the sections were incubated with the Alexa fluor 594-conjugated secondary antibodies. DAPI was detected as cell nuclei. Sections were observed under a fluorescence microscope FV10i confocal system (Olympus, Tokyo, Japan).

### Intra-epidermal nerve fiber density

The paraffin-embedded footpads were cut into 8-μm sections. The sections were incubated overnight at 4 °C with the primary antibody (anti-PGP9.5 antibody; Abnova, Taipei City, Taiwan) diluted 1:400 and subsequently stained using the Simplestain rat system (Nichirei) according to the manufacturer’s instructions. The negative control was performed by omitting the anti-PGP9.5 antibody. Each individual nerve fiber with branching in the epidermis from the dermis was counted. Intra-epidermal nerve fiber density (IENFD) was measured blindly in 10 fields from each section by three independent investigators under light microscopy (×200) and was expressed as epidermal nerve fiber number per length of epidermal basement membrane [23, 24].

### mRNA expression of cultured DPSCs and hindlimb skeletal muscles

RNA was extracted from cultured DPSCs and the frozen samples of hindlimb skeletal muscles using TRIzol Reagent (Invitrogen) according to the manufacturer’s instructions. Starting from 1 μg RNA, cDNA was synthesized using ReverTra Ace (Toyobo, Osaka, Japan) according to the manufacturer’s descriptions. Primers and probes for basic fibroblast growth factor (bFGF), vascular endothelial growth factor (VEGF), nerve growth factor (NGF), neurotrophin-3 (NT-3) and 18S rRNA and β2 microglobulin for the endogenous control were purchased from Taqman Gene Expression Assays (Applied Biosystems, Foster City, CA, USA). Polymerase chain reaction (PCR) products were visualized by agarose gel (Wako, Osaka, Japan)/ethidium bromide (Sigma). Real-time quantitative PCR (qRT-PCR) was performed and monitored by the ABI PRISM 7000 Sequence Detction System (Applied Biosystems). The relative quantity was calculated by the by the ΔΔCt method.

### Cryopreservation and thawing of DPSCs

After DPSCs were cultured at passage 3, the cells were harvested with trypsin-EDTA, resuspended with CELLBANKER (Amsbio, Cambridge, MA, USA) and frozen in a cryotube in a −80 °C freezer for 6 months.

When thawing, cells were immediately thawed in a 37 °C water bath and then added to 10 ml of the cultured medium containing fetal bovine serum. After the cells were washed three times with phosphate-buffered saline, thawed DPSCs were cultured in a plastic dish with α-MEM supplemented with 20 % fetal bovine serum and used as cryo-DPSCs.

### Adipogenic or osteogenic differentiation assay

For the differentiation assay, fresh-DPSCs and cryo-DPSCs at passage 3 to 4 were cultured for 2–3 weeks in the induction medium according to the manufacturer’s instructions (R&D Systems). Adipogenic differentiation medium contained 1 % h-insulin, 2 % L-glutamine, 10 % mesenchymal cell growth supplement, 0.5 % dexamethasone, 0.2 % indomethacin, and 0.1 % 3-isobutyl-methyl-xanthine. Adipogenic maintenance medium contained 1 % h-insulin, 2 % L-glutamine, and 10 % mesenchymal cell growth supplement. For the detection of adipogenic differentiation, we stained the lipid accumulation with oil red O (Polyscience, Warrington, PA, USA).

Osteogenic induction medium contained 0.5 % dexamethasone, 2 % L-glutamine, 0.5 % ascorbate, 1 % β-glycerophosphate and 10 % mesenchymal cell growth supplement. We detected osteogenic differentiation with alkaline phosphatase (ALP) activity using an ALP detection kit (Millipore).

### Proliferation assay

Fresh-DPSCs and cryo-DPSCs were seeded onto six-well plates at 1 × 10^4^ cells/well. The growth of DPSCs was determined by cell counts at days 3 and 5 after seeding. When counting, the cells were harvested with 0.05 % trypsin and were centrifuged at 1200 rpm for 5 min. The cell number was counted with a hemocytometer.

The proliferative potential of DPSCs was assessed by the MTT assay according to the manufacturer’s procedure (Cayman Chemical Company, Ann Arbor, MI, USA). After 3 and 5 days of culture, cells were seeded in 96-well culture plates (1 × 10^3^ cells/well) and incubated for another 6 h. Then MTT Reagent was added and the cells were incubated for 3 h to reveal the formazan dark crystals. Crystal-dissolving solution was added to each well and the absorbance was determined at 570 nm with a microplate reader.

### Statistical analysis

All results are expressed as mean ± SEM. Statistical analysis was performed by Student's *t*-test for comparisons between two groups and one-way analysis of variance followed by Bonferroni correction for multiple comparisons. The significant difference was indicated at the *P* < 0.05 level.

## Results

### Body weights and blood glucose concentrations of rats

At 12 weeks after STZ injection, diabetic rats showed severe hyperglycemia (normal rats: 95.5 ± 6.4 mg/dl; diabetic rats: 401.3 ± 43.7 mg/dl; *P* < 0.01) and significant reductions in body weight (normal rats: 510.5 ± 19.0 g; diabetic rats: 313.0 ± 23.9 g; *P* < 0.01).

### Characteristics of DPSCs

**Fig. 1:**
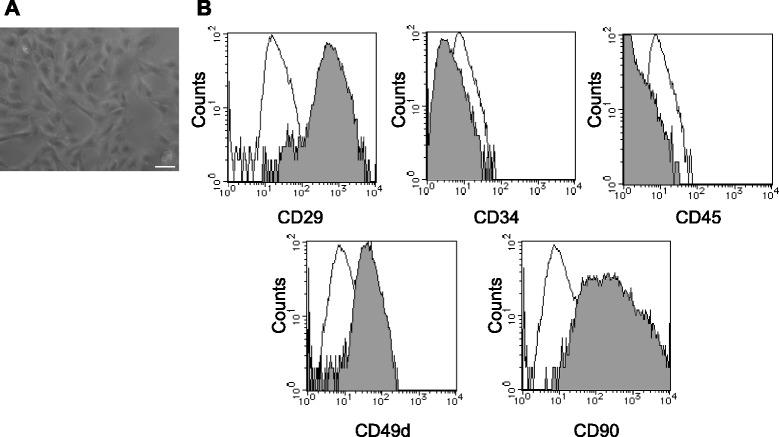
Morphology and characterization of fresh-DPSCs. **a** Cultured DPSCs observed with a phase contrast microscope. Bar = 100 μm. **b** Flow cytometric analysis of fresh-DPSCs. The expression of the surface markers was investigated with CD29, CD34, CD49d, CD45 and CD90. Open histograms, isotype controls; filled histograms, stained with the specific surface marker antibodies

Phase-contrast microscopic images of cultured DPSCs are shown in Fig. 1a. DPSCs were identified by their typical spindle-shape morphology. The surface markers of DPSCs showed high expression of CD29 and CD90, which are common stem cell markers in mesenchymal stem cells and DPSCs (Fig. 1b). DPSCs also showed relatively high expressions of CD49d, but lacked the expression of CD34 and CD45 [22]. The purity of DPSCs was achieved by passages and we found approximately 90 % purity after 3 passages. The differentiation ability of cultured DPSCs has already been confirmed in our previous paper [21].

### Fresh-DPSC transplantation ameliorates impaired MNCV, SNCV and SNBF in the diabetic rats

**Fig. 2:**
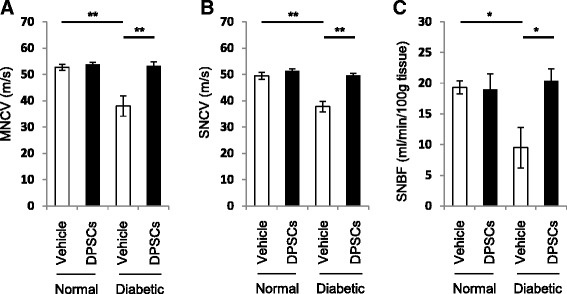
Effects of dental pulp stem cell (DPSC) transplantation on sciatic motor nerve conduction velocity (MNCV), sciatic sensory nerve conduction velocity (SNCV) and sciatic nerve blood flow (SNBF). DPSCs were transplanted into the unilateral hindlimb skeletal muscles 8 weeks after STZ injection. **a** MNCV. **b** SNCV. **c** SNBF. Diabetic rats showed significant reductions in MNCV, SNCV and SNBF. DPSC transplantation improved them to almost the same level as those of the normal rats. Results are expressed as means ±SEM (n = 4) . *P < 0.05, **P < 0.01

Eight-weeks after the STZ injection, we transplanted fresh-DPSCs into the unilateral hindlimb skeletal muscles. Neurophysiological measurements were performed at 4 weeks after DPSC transplantation. MNCV and SNCV in the vehicle-injected side of the diabetic rats were significantly reduced compared with those of the normal rats (Fig. 2a, b). DPSC transplantation significantly ameliorated both MNCV and SNCV in the DPSC-injected side compared with those in the vehicle-injected side of the diabetic rats (*P* < 0.01).

SNBF was also significantly decreased in the vehicle-injected side (*P* < 0.05), which was significantly augmented by the DPSC transplantation in the diabetic rats (*P* < 0.05) (Fig. 2c). Transplantation of DPSCs in the normal rats did not affect MNCV, SNCV, or SNBF.

### Fresh-DPSC transplantation increases capillary density of skeletal muscles in the diabetic rats

**Fig. 3:**
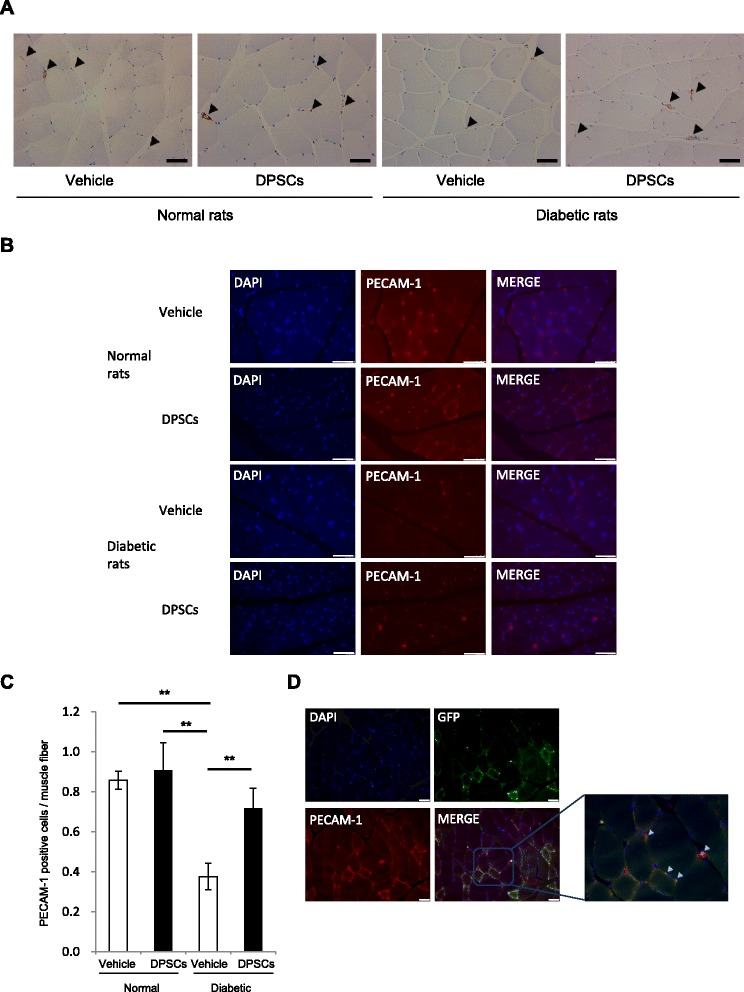
Capillaries in the soleus muscles. **a**, **b** Representative photomicrographs of the histological sections in the skeletal muscles of normal and diabetic rats. Capillaries were visualized by von Willebrand factor (vWF) (**a**) and PECAM-1 (**b**). Arrows indicate vascular endothelial cells stained by vWF. Bar = 50μm. **c** Quantitative analysis for capillary to muscle fiber ratio of the skeletal muscles of normal and diabetic rats. Results are expressed as means ± SEM (n = 4). **P < 0.01. **d** Differentiation of transplanted green fluorescent protein (GFP)-expressing dental pulp stem cells (DPSCs) into vascular endothelial cells in the skeletal muscles 4 weeks after the transplantation. DPSCs from GFP-expressing rats were transplanted into hindlimb skeletal muscles in the diabetic rats. Vascular endothelial cells were visualized by PECAM-1. Bar = 50 μm

The vasculatures in the soleus muscles were visualized by vWF immunostaining (Fig. 3a) and PECAM-1 immunofluorescence staining (Fig. 3b). Quantitative analyses revealed that the PECAM-1 positive endothelial cell to muscle fiber ratio was significantly reduced in the vehicle-injected side of diabetic rats compared with the normal rats (*P* < 0.01) (Fig. 3c). Transplantation of DPSCs significantly increased the PECAM-1 positive cell to muscle ratio in diabetic rats.

To confirm whether the increased capillaries are of host cell origin or from the differentiation into vascular endothelial cells from transplanted DPSCs, we transplanted GFP-DPSCs into diabetic rats. As shown in Fig. 3d, some of the PECAM-positive vascular endothelial cells are expressing GFP, indicating that transplanted DPSCs differentiated into vascular endothelial cells. Other PECAM-positive endothelial cells were of host origin.

### Transplanted DPSCs derived from GFP rats existed in the skeletal muscles without differentiation into adipocytes, osteoblasts, neuronal cells, or Schwann cells

Transplanted GFP-DPSCs existed around the muscle bundles in the skeletal muscles. Immunohistological staining revealed that transplanted GFP-DPSCs have not differentiated into adipocytes, osteoblasts, neurons or Schwann cells (Figure S1 in Additional file 1).

### Transplantation of fresh-DPSCs increases IENFD in the diabetic rats

**Fig. 4:**
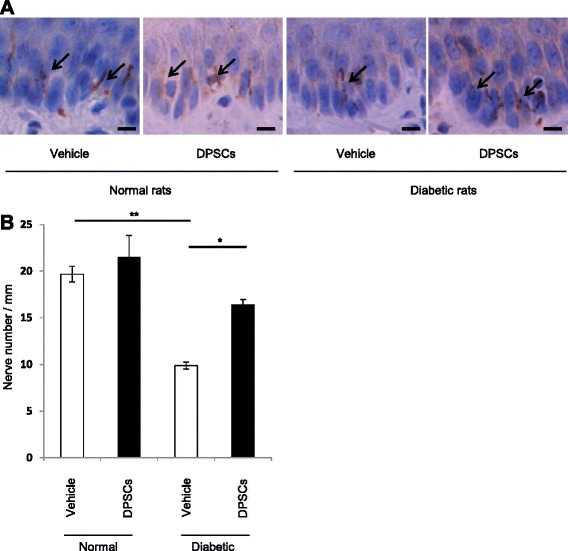
Intra-epidermal nerve fiber density (IENFD). **a** Representative photomicrographs of the histological sections of the footpads of normal and diabetic rats. Intra-epidermal nerve fibers (*arrows*) were detected by immunohistological staining for PGP9.5. Bar = 10μm. **b** Quantitative analysis for IENFD of the footpads of normal and diabetic rats. Results are expressed as means ± SEM (n = 4). *P < 0.05, **P < 0.01. *DPSC* dental pulp stem cell

To detect the sensory small nerve fiber damage in DPN, we analyzed IENFD at the footpad, which is established as a research tool for unmyelinated C and thinly myelinated A delta fibers and considered as a surrogate marker for small fiber neuropathy [23]. Intra-epidermal nerve fibers were visualized by PGP9.5 immunostaining (Fig. 4a). Quantitative analyses revealed that IENFD was significantly reduced by 50 % in the vehicle-injected side of diabetic rats compared with the normal rats (*P* < 0.01) (Fig. 4b), which was ameliorated by the transplantation of fresh-DPSCs (*P* < 0.05).

### Cultured DPSCs express mRNA expression of angiogenic and neurotrophic factors

**Fig. 5:**
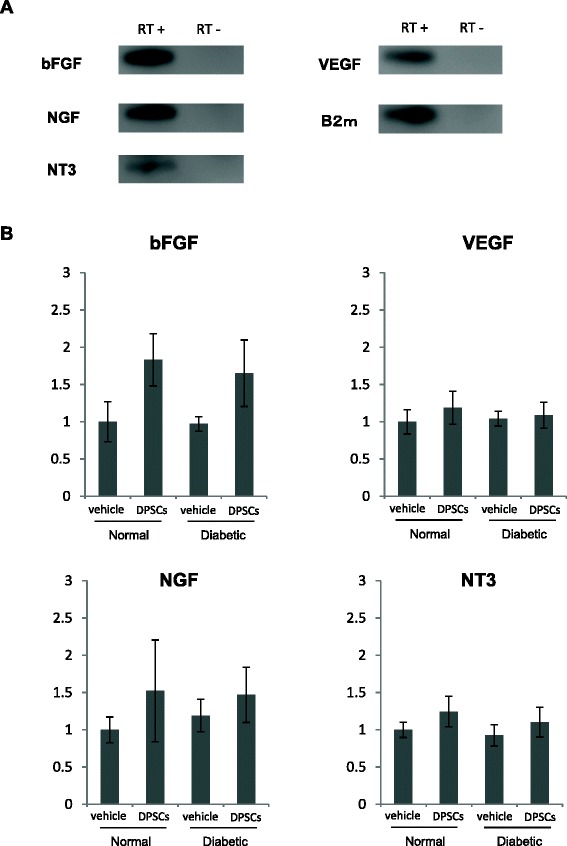
mRNA expressions of basic fibroblast growth factor (bFGF) vascular endothelial growth factor (VEGF), nerve growth factor (NGF) and neurotrophin-3 (NT-3) in cultured dental pulp stem cells (DPSCs) and hindlimb skeletal muscles. **a** mRNA levels of these factors in cultured DPSCs were evaluated by RT-PCR. The products were visualized by agarose gel/ethidium bromide. **b** mRNA expressions of these factors in the hindlimb skeletal muscles were measured by RT-PCR. The relative quantity was calculated by the by the ΔΔC^t^ method. Results are expressed as means ± SEM (n = 5)

To confirm whether cultured DPSCs express angiogenic and neurotrophic factors, we evaluated mRNA expression of VEGF, bFGF, NGF and NT-3 by RT-PCR. The products were visualized by agarose gel electrophoresis. As shown in Fig. 5a, DPSCs expressed all of these angiogenic and neurotrophic factors.

### Transplantation of fresh-DPSCs into skeletal muscles tends to affect mRNA expression of neurotrophic and angiogenic factors

To investigate whether DPSC transplantation affects mRNA expression in the transplanted skeletal muscles, we measured angiogenic and neurotrophic gene expressions in the skeletal muscles on the transplanted side and the vehicle-injected side. As shown in Fig. 5b, the mRNA expression of angiogenic and neurotrophic factors, especially bFGF and NGF, in the skeletal muscles tended to be increased on the transplanted side compared with those on the vehicle-injected side in both normal and diabetic rats, although they were not significant.

### Characteristics of cryo-DPSCs are similar to those of fresh-DPSCs

**Fig. 6:**
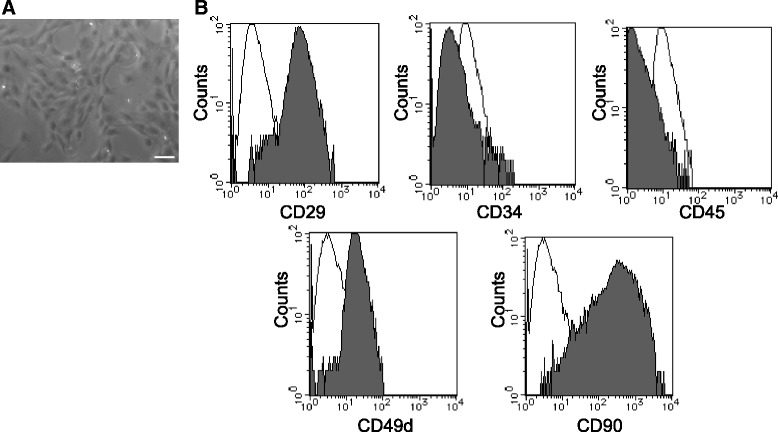
Morphology and identification of cryo-DPSCs. **a** DPSCs, which were thawed after cryopreservation, were cultured and expanded. Cryo-DPSCs were of similar shape as the fresh-DPSCs observed with the phase contrast microscope. Bar = 100 μm. **b** Flow cytometric analysis of cryo-DPSCs. The expression of surface markers was analyzed with CD29, CD34, CD49d, CD45 and CD90. Open histograms, isotype controls; filled histograms, stained with the specific surface marker antibodies

Cryo-DPSCs were thawed at 37 °C, re-cultured and expanded. The cryo-DPSCs showed the typical spindle-shaped morphology, which was the same as that of the fresh-DPSCs (Fig. 6a). The surface marker analysis revealed that cryo-DPSCs were positive for CD29, CD90 and CD49d and negative for CD34, CD45, which was the same pattern shown by the fresh-DPSCs (Fig. 6b).

### Differential and proliferative capability are preserved in cryo-DPSCs

**Fig. 7:**
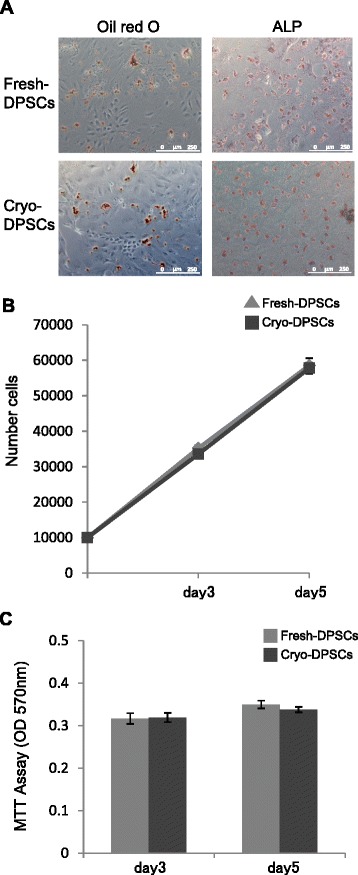
Differential potential and proliferative capacity of freshly isolated dental pulp stem cells (fresh-DPSCs) and cryopreserved dental pulp stem cells (cryo-DPSCs). **a** Adipogenic differentiation was observed by oil red O staining. Osteogenic differentiation was observed by alkaline phosphatase (ALP) activity. Bar = 250 μm. **b** The growth of fresh-DPSCs and cryo-DPSCs was measured as the cell number counted with a hemocytometer on day 3 and day 5 after seeding. **c** The proliferative activity of fresh-DPSCs and cryo-DPSCs was determined by the MTT assay on day 3 and day 5. Results are expressed as means ± SEM (n = 9)

We assessed the capacity of fresh-DPSCs and cryo-DPSCs to differentiate into adipocytes and osteoblasts. After adipogenic induction for 3 weeks, the cells were stained with oil red O for the detection of adipogenesis. Lipid cluster-positive cells were equally detected in both fresh-DPSCs and cryo-DPSCs (Fig. 7a). For the osteogenic induction, cells were cultured in the osteogenic induction medium for 2 weeks. ALP was identified at similar levels in fresh-DPSCs and cryo-DPSCs (Fig. 7a).

We examined the proliferative potential of fresh-DPSCs and cryo-DPSCs by cell count and MTT assay. The growth rate was not different between fresh-DPSCs and cryo-DPSCs by cell count (Fig. 7b). MTT assay showed that there was no significant difference in the proliferative potential between fresh-DPSCs and cryo-DPSCs on day 3 and day 5 (Fig. 7c).

### Impaired nerve function in the diabetic rats is ameliorated by the transplantation of cryo-DPSCs

**Fig. 8:**
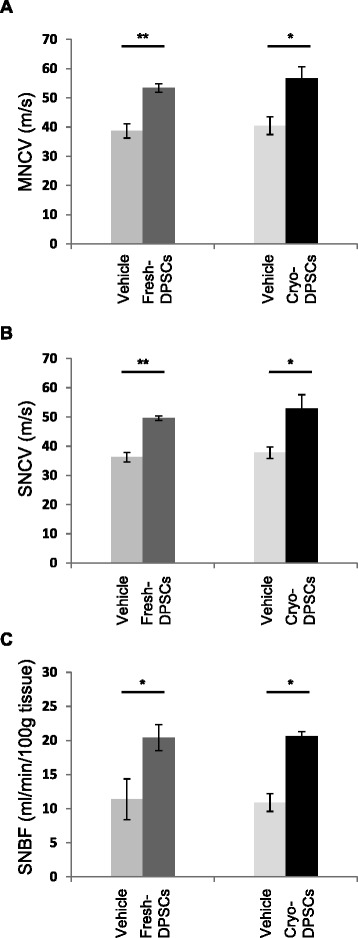
Comparison of the transplantation effects of freshly isolated dental pulp stem cells (fresh-DPSC) and cryopreserved dental pulp stem cells (cryo-DPSCs) on neurophysiological disorder. Transplantation of cryo-DPSCs significantly ameliorated the impaired motor nerve conduction velocity (MNCV) (**a**) and sensory nerve conduction velocity (SNCV) (**b**). These results were equal to those of fresh-DPSCs. Transplantation of cryo-DPSCs and fresh-DPSCs significantly increased the decreased sciatic nerve blood flow (SNBF) (**c**). Results are expressed as means ± SEM (n = 4). *P < 0.05, **P < 0.01

We evaluated the effects of fresh-DPSC transplantation and cryo-DPSC transplantation on the nerve functions in the diabetic rats. Eight weeks after STZ injection, fresh-DPSCs or cryo-DPSCs were transplanted into the unilateral hindlimb skeletal muscles in the diabetic rats and neurophysiological measurements were performed 4 weeks after transplantation. As shown in Fig. 8, the delayed MNCV and SNCV in the vehicle-injected side of the diabetic rats were significantly augmented by cryo-DPSC transplantation as well as fresh-DPSC transplantation (*P* < 0.05 and *P* < 0.01, respectively). The transplantation of fresh-DPSCs and cryo-DPSCs significantly increased SNBF compared with that in the vehicle injection side in diabetic rats (*P* < 0.05). The level of MNCV, SNCV, and SNBF on the transplanted side did not significantly differ between transplantations of fresh-DPSCs and cryo-DPSCs.

## Discussion

In this study, we revealed that the transplantation of cultured DPSCs into the hindlimb skeletal muscles ameliorated DPN in rats. We compared the fresh-DPSCs with the cryo-DPSCs and revealed that the cryo-DPSCs were not inferior in proliferative and differential ability compared with the fresh-DPSCs, and maintained their therapeutic potential for DPN.

DPN, an early onset and most frequent complication in diabetic patients, results from the impairment of nerve blood flow and from metabolic disorders [24]. Although recent anti-symptomatic drugs improve the symptoms of DPN, the establishment of more essential and effective treatments is needed. In this manuscript, we revealed that the transplantation of DPSCs into hindlimb skeletal muscles ameliorated the impaired MNCV, SNCV and SNBF in diabetic rats. The capillary to muscle fiber ratio of the diabetic rats was significantly reduced compared with that of normal rats, but was ameliorated by the transplantation of DPSCs. The diabetic rats also showed decreased IENFD on the footpad, and the transplantation of DPSCs increased IENFD on the footpad of the transplanted side. These results suggest that the transplantation of DPSCs, as well as the other types of mesenchymal stem cells, could have therapeutic potential for DPN with the improvement of both large and small size nerve fibers and nerve blood flow [14, 25].

Previous experimental and clinical studies indicated that vasodilator therapy has some promising effects on DPN [26, 27]. Here, we showed that DPSCs expressed angiogenic genes, such as bFGF and VEGF, and the DPSC transplantation into the hindlimb skeletal muscles increased microvasculatures in the skeletal muscles, accompanied with an increase in the sciatic nerve blood flow at the transplanted side. We used GFP-expressing DPSCs and confirmed that a small number of the transplanted DPSCs differentiated into vascular endothelial cells and directly participated in vasculogenesis, while other microvasculatures were of host origin. Although DPSCs can differentiate into many type of cells, most of which are mesodermal in origin such as osteoblast, adipocytes, and neurons [1, 21, 28, 29], we have not found any differentiation into osteoblast, adipocytes, neuronal cells, or Schwann cells. These results are consistent with the previous results of the therapeutic transplantation of other progenitor or stem cells [12, 14].

An important issue to be solved for autologous cell transplantation in diabetic patients is the functional disorder of progenitor and mesenchymal stem cells. Like other mature cells, the functions of progenitor and mesenchymal stem cells were impaired by hyperglycemia and aging [16–19]. The efficacy of progenitor and stem cell transplantation was affected by the activity of donor cells [18, 20, 30]. DPSCs are isolated from dental pulp and easily expanded in a culture condition. Dental pulp can be obtained from otherwise useless teeth extracted for orthodontic reasons. Previous investigations demonstrated that DPSCs isolated even 5 days after tooth extraction have high proliferative activity [31, 32]. Importantly, DPSCs were reported to maintain their proliferative and differential ability and keep their immunosuppressive activity even after cryopreservation [33, 34].

Our therapeutic strategy for DPSC transplantation to treat DPN is as follows: DPSCs are isolated from extracted teeth in young adults for orthodontic reasons. Obtained DPSCs are cryopreserved before use. Cryo-DPSCs are thawed and expanded in a culture condition as needed. Re-cultured DPSCs are transplanted for the treatment of DPN. To ensure the possibility of this strategy, we have investigated whether the transplantation of cryo-DPSCs ameliorated DPN in STZ-induced diabetic rats. We determined that the cryo-DPSC transplantation improved nerve conduction velocity and nerve blood flow as well as fresh-DPSC transplantation. We also confirmed that cryo-DPSCs expressed the same surface antigen pattern as the fresh-DPSCs, and kept their potential to differentiate into osteoblasts and adipocytes, which is consistent with the results of a previous investigation [35]. The proliferative activity measured by cell count and MTT assay was the same level in fresh-DPSCs and cryo-DPSCs. These results indicate that DPSCs maintain their function and therapeutic efficacy for the treatment of DPN after cryopreservation.

We have not evaluated for how long a single transplantation of DPSCs keeps the amelioration of DPN. We evaluated the effects of DPSC transplantation 4 weeks after transplantation in this study. The studies on the transplantation of other types of cells, such as mesenchymal stem cells and endothelial progenitor cells, demonstrated that the stem/progenitor cell transplantation ameliorated DPN 3 days to 12 weeks after transplantation, suggesting that the effects maintain for at least 12 weeks [13, 14, 36]. Further study is required on this issue.

## Conclusion

We demonstrated for the first time that the transplantation of fresh-DPSCs ameliorated nerve conduction velocity and nerve blood flow with an increase in IENFD in diabetic rats. Cultured DPSCs expressed angiogenic and neurotrophic factor genes, suggesting their multi-function. Cryo-DPSCs maintained the proliferative and differentiative potential at the same level as fresh-DPSCs and the effects of cryo-DPSC transplantation were not inferior to fresh-DPSC transplantation. Since DPSCs can be isolated from teeth extracted from young adults, the obtained DPSCs, which can be cryopreserved until needed, are not only youthful but also may be stem cells before the onset of diabetes. These findings suggest that DPSCs may be a promising cell source for the treatment of DPN.

## Additional file

Additional file 1: Figure S1.
**Transplanted GFP-expressing DPSCs were located in the skeletal muscles without differentiation into adipocytes, osteoblasts, neuronal cells, or Schwann cells**. DPSCs from GFP-expressing rats were transplanted into hindlimb skeletal muscles in the diabetic rats. To analyze the differentiation of transplanted DPSCs, The skeletal muscles were stained with the primary antibody against FABP for adipocytes, osteocalcin for osteoblasts, neuronal nuclei (NeuN) for neurons and glial fibrillary acidic protein for Schwann cells. (PPTX 409 kb)

## Abbreviations

ALP: Alkaline phosphatase
bFGF: Basic fibroblast growth factor
cryo-DPSC: Cryopreserved dental pulp stem cell
DAPI: 4’-6-Diamidino-2-phenylindole
DPN: Diabetic polyneuropathy
DPSC: Dental pulp stem cell
FACS: Fluorescence activated cell sorting
fresh-DPSC: Freshly isolated dental pulp stem cell
GFP: Green fluorescent protein
IENFD: Intra-epidermal nerve fiber density
MNC: Mononuclear cell
MNCV: Motor nerve conduction velocity
NeuN: Neuronal nuclei
NGF: Nerve growth factor
NT-3: Neurotrophin-3
PCR: Polymerase chain reaction
PGP9.5: Protein gene product 9.5
qRT-PCR: Real-time quantitative polymerase chain reaction
SNBF: Sciatic nerve blood flow
SNCV: Sensory nerve conduction velocity
STZ: Streptozotocine
VEGF: Vascular endothelial growth factor
vWF: von Willebrand factor
α-MEM: Alpha modification of the Eagle’s medium
