# Supplementary material for: Transplantation of cultured dental pulp stem cells into the skeletal muscles ameliorated diabetic polyneuropathy: therapeutic plausibility of freshly isolated and cryopreserved dental pulp stem cells
Source: Stem Cell Res Ther. 2015 Sep 7;6(1):162. doi: 10.1186/s13287-015-0156-4 (PMC4562193; doi:10.1186/s13287-015-0156-4)
Supplement: Additional file 1: Figure S1. — Transplanted GFP-expressing DPSCs were located in the skeletal muscles without differentiation into adipocytes, osteoblasts, neuronal cells, or Schwann cells. DPSCs from GFP-expressing rats were transplanted into hindlimb skeletal muscles in the diabetic rats. To analyze the differentiation of transplanted DPSCs, The skeletal muscles were stained with the primary antibody against FABP for adipocytes, osteocalcin for osteoblasts, neuronal nuclei (NeuN) for neurons and glial fibrillary acidic protein for Schwann cells. (PPTX 409 kb) [file 13287_2015_156_MOESM1_ESM.pptx]

## Slide 1
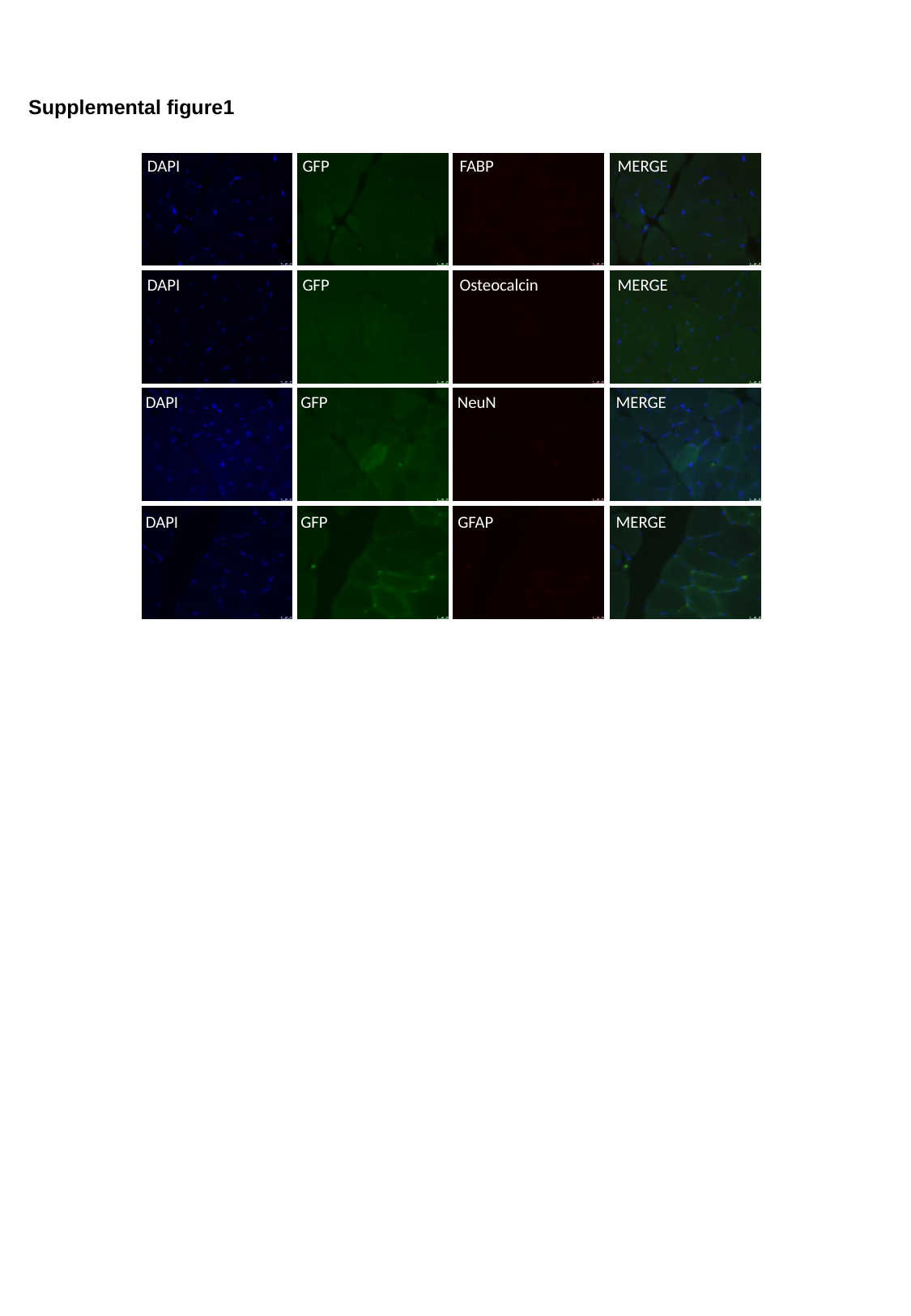

Supplemental figure1
DAPI
GFP
FABP
MERGE
DAPI
GFP
Osteocalcin
MERGE
DAPI
GFP
NeuN
MERGE
DAPI
GFP
GFAP
MERGE
